# Relationship between Plasma Ferritin Level and Siderocyte Number in Splenectomized **β**-Thalassemia/HbE Patients

**DOI:** 10.1155/2012/890471

**Published:** 2012-10-24

**Authors:** A. Tripatara, N. Srichana, P. Lamool, S. Amnuaykan, P. Hongart, A. Jetsrisuparb

**Affiliations:** ^1^Division of Medical Technology, Faculty of Associated Medical Sciences, Khon Kaen University, Khon Kaen 40002, Thailand; ^2^Center for Research and Development of Medical Diagnostic Laboratories, Khon Kaen University, Khon Kaen 40002, Thailand; ^3^Clinical Chemistry Unit, Srinagarind Hospital, Khon Kaen University, Khon Kaen 40002, Thailand; ^4^Department of Pediatrics, Faculty of Medicine, Khon Kaen University, Khon Kaen 40002, Thailand

## Abstract

*Introduction*. In iron overload status, excess iron deposits in reticuloendothelial cells and tissues and can be detected using Prussian blue staining. The aim of this paper was to investigate the relationship between siderocyte numbers and plasma ferritin levels (a practically standard marker of iron overload) in the blood of the splenectomized and nonsplenectomized *β*-thalassemia/HbE patients, who are at risk of iron overload. *Methods*. EDTA blood samples from 64 patients with 35 splenectomized and 29 nonsplenectomized *β*-thalassemia/HbE patients, who received regular blood transfusions, and 20 normal individuals were investigated for siderocyte numbers, plasma ferritin levels, and complete blood counts. *Results*. The average percent siderocytes in splenectomized and nonsplenectomized *β*-thalassemia/HbE patients were 11.5% and 0.08%, respectively, and plasma ferritin levels of 2,332 *μ*g/L and 1,279 *μ*g/L, respectively. Percent siderocytes showed a good correlation with plasma ferritin levels only in splenectomized patients (*r* = 0.69, *P* < 0.001). A receiver operating curve analysis from splenectomized patients' data indicated that siderocytes at 3% cut-off are the best predictor for plasma ferritin level ≥1,000 **μ**g/L with 92.9% sensitivity and 42.9% specificity. *Conclusion*. Circulating siderocyte numbers can be used as a screening test for the assessment of the iron overload in splenectomized *β*-thalassemia/HbE patients in the place where serum ferritin is not available.

## 1. Introduction

Siderocyte is defined as red blood cell containing nonhemoglobin iron, which is not detectable in the blood circulation of a healthy person or a patient with normal spleen function [1]. Siderocytes may be found in peripheral blood of splenectomized patients, who have impaired iron metabolic disease, such as sideroblastic anemia or hemolytic anemia (namely, thalassemia), which result in iron accumulation [2].

Prussian blue staining is a simple method for detecting nonhemoglobin iron in cells, bone marrow, and tissues [3]. It is useful for diagnosis of iron overload, although at present it is not often employed for this purpose. However, by using Prussian blue staining iron deposition in red blood cells can be readily visualized, appearing as blue-green granules in the red blood cell cytoplasm [4].

Thalassemia is an autosomal genetic disease leading to anemia and remains one of the major health problems in Southeast Asia and other parts of the world where malaria is or has been endemic [5]. In severe cases, in order to improve survival and quality of life of these patients, multiple blood transfusions accompanied by iron chelation are required [6]. However, regular transfusions can lead to severe iron accumulation in cells and tissues, and thus the patient's body iron status must be monitored in order to provide a proper management of the iron overload status [7, 8].

A variety of tests are currently used to assess iron overload, including serum ferritin level, computed tomography (CT), magnetic resonance imaging (MRI), and liver iron content (from biopsy) [9]. Among these procedures, serum ferritin level is a commonly used measurement as it is minimally invasive, inexpensive, and widely available and can be performed frequently allowing regular monitoring, and the values correlate with total body iron store [8, 10]. Serum ferritin levels consistently >1000 *μ*g/L are indicative of iron overload [7, 10, 11].

It is recommended that iron chelation should be given to thalassemic patients who receive regular blood transfusions when ferritin levels rise above 1,000 *μ*g/L [6, 8]. It has not been demonstrated hitherto whether the numbers of siderocytes in the blood of splenectomized thalassemic patients have any correlation with body iron store detected in the form of serum ferritin and thereby be helpful in monitoring iron overload status of such patients.

## 2. Materials and Methods

### 2.1. Blood Samples

EDTA blood samples from 64 *β*-thalassemia/HbE patients (35 splenectomized; 29 nonsplenectomized), who came for regular followups at Srinagarind hospital, Khon Kaen University, Thailand, and 20 healthy individuals were recruited into the study. The duration from the last blood transfusion to the time the blood samples were being collected was at least 3 weeks. This work was approved by the Khon Kaen University Ethics Committee for Human Research (approval no. HE532278).

### 2.2. Laboratory Measurements

Plasma ferritin was measured using an immunoturbidimetric assay (Cobas 6000, Roche Diagnostics). Complete blood count was determined by KX1 Hematology analyzer (Sysmex Corporation). Siderocyte counting was performed on a thin blood film that was fixed with absolute methanol and stained with the solution of 2% K_4_Fe(CN)_6_ and 2% HCL at ratio 2 : 1 and counterstained with 1% Eosin [12] and examined under a light microscope (Nikon ECLIPSE 80*i*) connected to a digital camera and a computer monitor (ASUSTeK Computer Inc). Siderocytes were counted per 1,000 red blood cells and expressed as percent.

### 2.3. Statistical Analysis

Results are expressed as mean ± SD. The comparison between groups was performed using *t*-test or the Mann-Whitney *U* test. Pearson correlation was employed for analysis of the relationship between numbers of siderocytes and serum ferritin levels. For determining the optimal cut-off value for predicting iron overload, a receiver operator curve (ROC) was performed. *P*  value < 0.05 is considered statistically significant. All calculations were conducted with SPSS 17.0 statistical software.

## 3. Results

### 3.1. Hematological Parameters

Hematological parameters of *β*-thalassemia/HbE patients (hemoglobin, red blood cells, MCV, MCH, MCHC, and RDW) are shown in Table 1. There were no statistical differences in these parameters between splenectomized and nonsplenectomized groups, except MCV.

### 3.2. Relationship between Percent Siderocytes and Plasma Ferritin Levels

**Figure 1:**
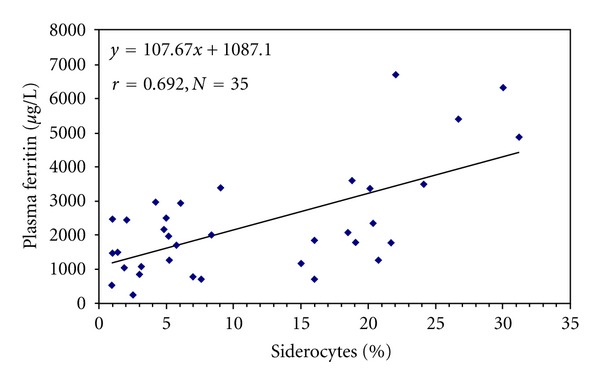
Relationship between plasma ferritin levels and percent siderocyte in splenectomized *β*-thalassemia/HbE patients. (A thin blood film was made, fixed in absolute methanol, and stained with Prussian blue staining. Siderocytes were counted per 1,000 red blood cells under light microscope connected to a digital camera and computer monitor. Plasma ferritin was measured using an immunoturbidimetric assay.)

Less than 1% of siderocytes (mean ± SD = 0.08 ± 0.16; *n* = 29) were presented in blood of nonsplenectomized *β*-thalassemia/HbE patients, whereas percent siderocytes in splenectomized subjects ranged from 0.93 to 31.23 (mean ± SD = 11.5 ± 9.4; *n* = 35) (*P* < 0.001). No siderocyte was found in healthy controls. Plasma ferritin levels ranged from 276–6,700 *μ*g/L (mean ± SD = 2,332 ± 1570) and 320–3,750 *μ*g/L (mean ± SD = 1,279 ± 811) (*P* = 0.001) in splenectomized and nonsplenectomized *β*-thalassemia/HbE groups, respectively. (Plasma ferritin in normal controls was 36 ± 11 *μ*g/L.) There was a significant correlation between percent siderocytes and plasma ferritin levels in splenectomized patients (*r* = 0.69, *P* < 0.001) (Figure 1).

### 3.3. Sensitivity and Specificity in Predicting Iron Overload from Percent Siderocytes

**Figure 2:**
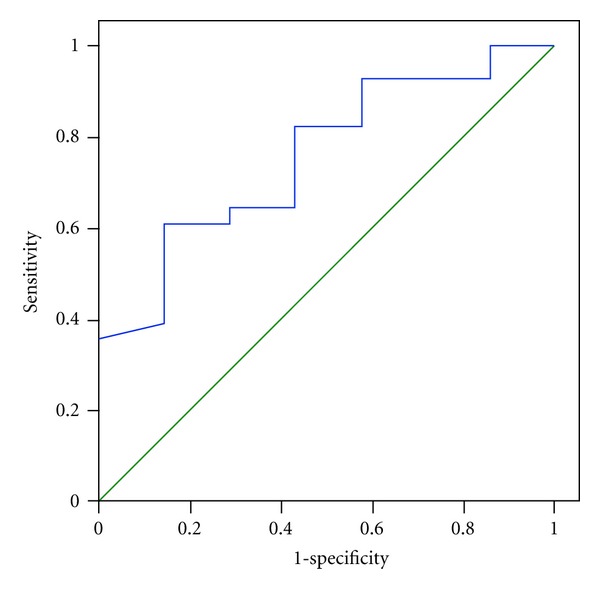
A receiver operating characteristic curve of siderocytes in splenectomized *β*-thalassemia/HbE patients.

We considered a ferritin level of >1,000 *μ*g/L as an indicator of iron overload and iron chelators should be given. The area under the ROC in the diagnosis of iron overload was 0.758 (*P* = 0.037) (Figure 2). If percent siderocyte ≥1 was selected as the cut-off value, sensitivity and specificity were 75% and 57.1%, respectively. However, the best cut-off value of percent siderocyte for detecting iron overload was ≥3, with 92.9% sensitivity and 42.9% specificity.

## 4. Discussion

Ferritin is the main source of stored iron whereas hemosiderin is described as degraded form of ferritin [13] appearing as blue intracellular granules that are large enough to be viewed by a light microscopy [1]. Iron was taken from plasma to cytosol of young erythroid cells in the bone marrow for heme synthesis via transferrin-transferrin receptor pathway [14]. Excess iron was stored in the form of ferritin in the cytosol [15] and mitochondria. [16]. Thalassemia patients not only have impaired hemoglobin biosynthesis, but also have excess iron uptake that is incorporated into ferritin and remains inside the red cells. Ferritin is not usually stained by Prussian Blue reaction due to its soluble property. It is not known how ferritin in mitochondria would be degraded to form a hemosiderin-like material; however, in the cytosol, this may occur by lysosomal degradation [17].

Several reports have suggested that in transfusion-dependent patients, such as in thalassemia, plasma ferritin levels >1,000 *μ*g/L are indicative of iron overload, and such patients require iron chelating therapy in order to prevent organ damage from iron toxicity [6–8, 10, 11]. As there was a significant correlation between percent siderocytes and plasma ferritin levels in splenectomized *β*-thalassemia/HbE patients, using ROC approach, a cut-off value of siderocytes ≥3% produced the best predictor of >90% sensitivity but <50% specificity.

Thus, at least for splenectomized *β*-thalassemia/HbE patients, determining percent siderocytes from blood smears provides a simple and an inexpensive method for screening and monitoring iron overload in such patients who are undergoing regular blood transfusions with limited facilities and economic resources. This technique is suitable in laboratories with limited resources and is useful in those regions where economic problem does not permit to evaluate serum ferritin levels.

## Figures and Tables

**Table 1:** Hematologic parameters of *β*-thalassemia/HbE patients.

	Splenectomized patients (*N* = 35)	Nonsplenectomized patients (*N* = 29)	*P* value
Hemoglobin (g/dL)	7.4 ± 1.1	7.5 ± 1.0	0.676
RBC count (×10^6^/*μ*L)	3.2 ± 0.7	3.5 ± 0.5	0.119
MCV (fL)	79.6 ± 10.5	67.3 ± 4.6	0.011
MCH (pg)	23.4 ± 4.3	21.4 ± 1.8	0.133
MCHC (g/dL)	29.3 ± 2.6	31.7 ± 1.5	0.136
RDW (%)	26.7 ± 5.9	30.1 ± 3.8	0.129

Data presented as mean ± SD. For normal individuals, MCV = 86.6 ± 1.1 fL, MCH = 27.3 ± 0.6 pg, MCHC = 31.5 ± 0.5 g/dL, RDW = 12.8 ± 0.6%, RBC = 4.4 ± 0.2 × 10^6^/*μ*L, Hb = 12.5 ± 0.8 g/dL.

## References

[1] Shinton NK (2008). *Desk Reference for Hematology*.

[2] Crosby WH (1959). Normal functions of the spleen relative to red blood cells: a review. *Blood*.

[3] Greer JP, Foerster J, Rodgers GM, Paraskevas F, Arber DA, Mean RT (2009). *Wintrobe's Clinical Hematology*.

[4] Kurth D, Deiss A, Cartwright GE (1969). Circulating siderocytes in human subjects. *Blood*.

[5] Fucharoen S, Winichagoon P (1992). Thalassemia in Southeast Asia: problems and strategy for prevention and control. *Southeast Asian Journal of Tropical Medicine and Public Health*.

[6] The Thalassemia International Federation (2007). *Guidelines for the Clinical Management of Thalassemia*.

[7] Perifanis V, Economou M, Christoforides A, Koussi A, Tsitourides I, Athanassiou-Metaxa M (2004). Evaluation of iron overload in *β*-thalassemia patients using magnetic resonance imaging. *Hemoglobin*.

[8] Porter JB (2001). Practical management of iron overload. *British Journal of Haematology*.

[9] Borgna-Pignatti C, Castriota-Scanderbeg A (1991). Methods for evaluating iron stores and efficacy of chelation in transfusional hemosiderosis. *Haematologica*.

[10] Olivieri NF, Brittenham GM (1997). Iron-chelating therapy and the treatment of thalassemia. *Blood*.

[11] Morrison ED, Brandhagen DJ, Phatak PD (2003). Serum ferritin level predicts advanced hepatic fibrosis among U.S. patients with phenotypic hemochromatosis. *Annals of Internal Medicine*.

[12] Turgeon ML (2005). *Clinical Hematology Theory and Procedures*.

[13] Napier I, Ponka P, Richardson DR (2005). Iron trafficking in the mitochondrion: novel pathways revealed by disease. *Blood*.

[14] Hentze MW, Muckenthaler MU, Andrews NC (2004). Balancing acts: molecular control of mammalian iron metabolism. *Cell*.

[15] Harrison PM, Arosio P (1996). The ferritins: molecular properties, iron storage function and cellular regulation. *Biochimica et Biophysica Acta*.

[16] Drysdale J, Arosio P, Invernizzi R (2002). Mitochondrial ferritin: a new player in iron metabolism. *Blood Cells, Molecules & Diseases*.

[17] Radisky DC, Kaplan J (1998). Iron in cytosolic ferritin can be recycled through lysosomal degradation in human fibroblasts. *Biochemical Journal*.

